# Arsenicum-album

**Published:** 1895-12

**Authors:** 


### HOMŒOPATHIC PHYSICIAN,

A MONTHLY JOURNAL OF

HOMŒOPATHIC MATERIA MEDICA AND CLINICAL MEDICINE.

"If our school ever gives up the strict inductive method of Hahnemann, we are lost, and deserve only to be mentioned as a caricature in the history of medicine."—CONSTANTINE HERING.

Vol. XV. DECEMBER, 1895. No. 12.

### EDITORIAL.

ARSENICUM-ALBUM.—Under this remedy we find profuse leucorrhœa which is acrid and corroding, and dropping out when standing.

Arsenicum has hoarseness in the morning, hoarseness with coryza, and hoarseness with roughness of the throat. The patient talks fast on account of the dyspnœa. He has sense of chilliness or coldness in the chest. There is also coldness in the abdomen. Sulphur also has coldness in the chest. The Arsenicum patient also has cough and dyspnœa at night, obliging him to sit up in bed, and hoarseness in the morning.

Arsenicum has pain and soreness in upper third of right lung or all the apex, and occasionally lower part of left lung. This is Dr. Guernsey's key-note for Arsenic.

Arsenicum has cough from walking in cold air. Phosphorus also has cough, worse in cold air. Arsenicum, there is arrest of breathing after cough, as if from constriction of the chest.

Arsenicum has gray, greenish expectoration. Stannum has greenish expectoration of a disagreeable sweet taste and very profuse. Sometimes the expectoration has a salty taste. Quite a number of remedies have greenish expectoration, for which the student should consult a repertory.

Arsenicum has yellow spots on the chest, and Sepia has claret-colored spots. Sepia has a yellow saddle across the nose, and Sulphur has a red one. These indications, properly remembered, will often lead up to the right remedy.

Under Arsenicum, when lying on the back the heart beats more quickly and with violence.

Arsenicum has stiffness of the back of the neck. This remedy also has drawing pain between the shoulder-blades, compelling one to lie down. It has stiffness of the neck, as just stated, as if from overlifting, or as if from bruising. There is painfulness of arm on which one lies at night, and painful swelling of the hands, with coldness. The rheumatic pains in the upper arm of the Arsenicum patient are similar to Rhus-tox. Arsenicum has aching of the limbs, waking him from sleep, especially in drunkards. The finger-joints are painful on moving them. There is cramp or rigidity of the fingers; fingers stiff and numb; tearing in the tibia, principally the right one. Phosphorus has swelling of the tibia. Kali-hydriodicum has painful nodes along the ridge of the tibia. This is another one of Dr. Guernsey's key-notes.

Arsenicum has tearing and stinging, apparently in the periosteum of the right tibia, extending down the leg into the big toe. There is paralysis of the legs; violent tearing in arms and legs. It is impossible to lie on the painful side; relieved only by constant motion. There is coldness of the legs and feet, sometimes with cold perspiration. The patient can't get warm. Swelling of legs, with excruciating pain; restlessness of legs at night, preventing sleep; they have to be moved all the time. Cramps in legs at night; the skin of the soles of the feet is thick, hard, without sensation, and full of cracks. Arsenicum has ulcers on the soles of the feet and toes. Bryonia has ulcers with coldness. Arsenicum has ulcers on the heels, with bloody pus. Natrum-carbonicum has ulcers on heels from rubbing of boots. Dr. Frederic Preston has called attention to Lamium-album and Allium-cepa for this trouble of the heel. In Lippe's *Materia Medica,* Lamium has "blister on the heel from slight rubbing, afterward bursting and changing to an ulcer, with smarting and biting." Allium-cepa has "soreness, especially of the heel where the skin is rubbed off by the shoes." This also is from Lippe's *Materia Medica.*

Arsenicum has paralysis of the legs. There is total inability to raise them. This is similar to Nux-vomica. Arsenicum has trembling of limbs in drunkards. This is similar to Lachesis and Sulphur. Arsenicum has sensation of breaking down in the knees on going up-stairs.

Arsenicum has fainting from weakness, followed by aversion to least motion, and aggravation of debility by motion. The patient is troubled with coldness from sitting still. Numbers of remedies have fainting from weakness. Dr. Lippe mentioned especially three: Gelsemium, Pulsatilla, and Sepia.

With the debility the Arsenicum patient has much depression of spirits, especially in phthisis, with moaning and weeping.

Starting of the limbs when on the point of falling to sleep is not only a symptom of Arsenic, but of Belladonna as well.

In connection with this symptom of starting, the Editor cannot refrain from adding a few of his own notes taken from his private note book:

Argentum-metallicum, starting like an electrical shock on falling to sleep, waking her up. The shocks may be of whole body or single limb.

Cina, starting when falling to sleep; screams, turns over, and kicks covers off.

Arum-triphyllum, Belladonna, Mercurius, starting when falling to sleep.

Belladonna, starting when falling to sleep, feet jerked upward, and head forward.

Apis, starting at any noise when falling to sleep, or when some one enters the room.

Arsenic, Bell., Bry., Caust., Ign., Lyc., Nux-v., Op., Puls., Sepia, all have starting of limbs on falling to sleep.

Arum-triphyllum, starting up as if from fright on falling to sleep.

Aconite, wakens with a start from dreams.

Belladonna, starting in fright at the approach of others.

Stramonium, starting in fright on first waking from sleep at the approach of the nurse, or with shrinking from the first object the child sees. This is Dr. Guernsey's keynote.

Carbo-veg., starting from anxious dreams.

